# Bone marrow graft versus peripheral blood graft in haploidentical hematopoietic stem cells transplantation: a retrospective analysis in1344 patients of SFGM-TC registry

**DOI:** 10.1186/s13045-023-01515-4

**Published:** 2024-01-07

**Authors:** Claire Lacan, Jérôme Lambert, Edouard Forcade, Marie Robin, Patrice Chevallier, Sandrine Loron, Claude-Éric Bulabois, Corentin Orvain, Patrice Ceballos, Etienne Daguindau, Amandine Charbonnier, Yves Chalandon, Marc Bernard, Célestine Simand, Marie-Thérèse Rubio, Pascal Turlure, Johan Maertens, Anne Huynh, Michael Loschi, Jacques-Olivier Bay, Gaëlle Guillerm, Mustafa Alani, Cristina Castilla-Llorente, Xavier Poiré, Sylvain Chantepie, Natacha Maillard, Yves Beguin, Ambroise Marçais, Jérôme Cornillon, Jean-Valère Malfuson, Sébastien Maury, Nathalie Meuleman, Alban Villate, Mohammed-Amine Bekadja, Anouk Walter-Petrich, Nathalie Jacque, Micha Srour, Raynier Devillier, Stéphanie Nguyen

**Affiliations:** 1 Clinical Hematology Unit, Groupe Hospitalier Pitié-Salpêtrière, APHP, 47-83 Bd de l’Hôpital, 75013 Paris, France; 2 Institut national de la santé et de la recherche médicale (INSERM), U1153 CRESS, Paris, France; 3 Service de Biostatistique et Information Médicale, Hôpital Saint Louis, APHP, Paris, France; 4 Clinical Hematology Unit, Centre Hospitalier Universitaire de Bordeaux, Bordeaux, France; 5 Clinical Hematology Unit, Hôpital Saint Louis, APHP, Paris, France; 6 Clinical Hematology Unit, Centre Hospitalier Universitaire de Nantes, Nantes, France; 7 Clinical Hematology Unit, Hôpital Lyon Sud, HCL, Lyon, France; 8 Clinical Hematology Unit, Centre Hospitalier Universitaire de Grenoble, Grenoble, France; 9 Clinical Hematology Unit, Centre Hospitalier Universitaire d’Angers, Angers, France; 10 Clinical Hematology Unit, Centre Hospitalier Universitaire de Montpellier, Montpellier, France; 11 Clinical Hematology Unit, Centre Hospitalier Universitaire de Besançon, Besançon, France; 12 Clinical Hematology Unit, Centre Hospitalier Universitaire d’Amiens, Amiens, France; 13 Clinical Hematology Unit, Hôpitaux Universitaires de Genève, Geneva, Switzerland; 14 Clinical Hematology Unit, Centre Hospitalier Universitaire de Rennes, Rennes, France; 15 Clinical Hematology Unit, Institut de Cancérologie Strasbourg Europe, Strasbourg, France; 16 Clinical Hematology Unit, Centre Hospitalier Universitaire de Nancy, Nancy, France; 17 Clinical Hematology Unit, Centre Hospitalier Universitaire Dupuytren, Limoges, France; 18 Department of Hematology, UZ Leuven, Leuven, Belgium; 19 Clinical Hematology Unit, Oncopôle, Toulouse, France; 20 Clinical Hematology Unit, Centre Hospitalier Universitaire de Nice, Nice, France; 21 Clinical Hematology Unit, Centre Hospitalier Universitaire de Clermont-Ferrand, Clermont-Ferrand, France; 22 Clinical Hematology Unit, Centre Hospitalier Universitaire de Brest, Brest, France; 23 Clinical Hematology Unit, Centre Henri Becquerel, Rouen, France; 24 Clinical Hematology Unit, Institut Gustave Roussy, Villejuif, France; 25 Clinical Hematology Unit, Clinique Universitaire Saint Luc, Leuven, Belgium; 26 Clinical Hematology Unit, Centre Hospitalier Universitaire de Caen, Caen, France; 27 Clinical Hematology Unit, Centre Hospitalier Universitaire de Poitiers, Poitiers, France; 28 Clinical Hematology Unit, Centre Hospitalier Universitaire de Liège and University of Liège, Liège, Belgium; 29 Clinical Hematology Unit, Hôpital Necker-Enfants Malades, APHP, Paris, France; 30 Clinical Hematology Unit, Centre Hospitalier Universitaire de Saint Etienne, Saint Etienne, France; 31 Clinical Hematology Unit, Hôpitaux d’Instruction des Armées Percy, Clamart, France; 32 Clinical Hematology Unit, Hôpital Henri Mondor, APHP, Créteil, France; 33 Clinical Hematology Unit, Institut Jules Bordet, Brussels, Belgium; 34 Clinical Hematology Unit, Hôpital Bretonneau, Tours, France; 35 Clinical Hematology Unit, Clinic of Hematology and Cell Therapy, EHU 1St November, Oran, Algeria; 36 Clinical Hematology Unit, Centre Hospitalier Universitaire de Lille, Lille, France; 37 Clinical Hematology Unit, Institut Paoli Calmette, Marseille, France

**Keywords:** Haploidentical hematopoietic stem cell transplantation, Graft source, Bone marrow, Peripheral stem cell, Anti-thymoglobulin

## Abstract

The use of peripheral blood (PB) or bone marrow (BM) stem cells graft in haploidentical hematopoietic stem cell transplantation with post-transplant cyclophosphamide (PTCy) for graft-versus-host disease (GVHD) prophylaxis remains controversial. Moreover, the value of adding anti-thymoglobulin (ATG) to PTCy is unknown. A total of 1344 adult patients received an unmanipulated haploidentical transplant at 37 centers from 2012 to 2019 for hematologic malignancy. We compared the outcomes of patients according to the type of graft, using a propensity score analysis. In total population, grade II–IV and III–IV acute GVHD (aGVHD) were lower with BM than with PB. Grade III–IV aGVHD was lower with BM than with PB + ATG. All outcomes were similar in PB and PB + ATG groups. Then, in total population, adding ATG does not benefit the procedure. In acute leukemia, myelodysplastic syndrome and myeloproliferative syndrome (AL-MDS-MPS) subgroup receiving non-myeloablative conditioning, risk of relapse was twice greater with BM than with PB (51 vs. 22%, respectively). Conversely, risk of aGVHD was greater with PB (38% for aGVHD II–IV; 16% for aGVHD III–IV) than with BM (28% for aGVHD II–IV; 8% for aGVHD III–IV). In this subgroup with intensified conditioning regimen, risk of relapse became similar with PB and BM but risk of aGVHD III–IV remained higher with PB than with BM graft (HR = 2.0; range [1.17–3.43], *p* = 0.012).

**Supplementary Information:**

The online version contains supplementary material available at 10.1186/s13045-023-01515-4.

To the Editor,

Haploidentical hematopoietic stem cell transplantations account for a quarter of allogeneic HSCT worldwide [1–3]. There is no consensus on the use of bone marrow (BM) versus peripheral blood (PB) stem cells and on the value of adding anti-thymoglobulin (ATG) to post-transplant cyclophosphamide (PTCy) [4–7]. We report the experience of the Société Francophone de Greffe de Moelle et de Thérapie Cellulaire (SFGM-TC) retrospectively comparing the use of BM versus PB versus PB + ATG in patients who received T cell repleted graft from a haploidentical familial donor with PTCy between 2012 and 2019 in 37 centers. In addition, subgroup analyses of acute leukemia, myelodysplastic syndrome and myeloproliferative syndrome (AL-MDS-MPS) were performed according to conditioning intensity. A propensity score was used to make these different groups comparable (Additional files 1, 4).

## Findings

A total of 1344 patients were reported, including 371 BM, 776 PB without ATG and 197 received PB with ATG. The median age at transplant was 56 (IQR, 40.7–63.7). Patients were treated for AL (56%) or another myeloid (24%) or lymphoid (20%) malignancies. Myeloablative conditioning (MAC) regimen was more frequently used with BM (*p* < 0.05). PB + ATG patients had higher disease risk index score than other patients (*p* < 0.0001). Median follow-up was 28.7 months (Additional file 3: Table S1).

In the total population, engraftment and platelet reconstitution were similar between the three groups (Additional file 3: Table S2).

The cumulative incidence (CI) of 3-month aGVHD II–IV and aGVHD III–IV was 27.9%, 38.3%, 34.2%, and 7.7%, 15.9%,17.3% with BM, PB and PB + ATG, respectively (*p* < 0.05). The CI of two-year extensive chronic GVHD (cGVHD) was 11.8% without difference in the groups. After adjustments, the risk of aGVHD was lower with BM than with PB grafts. The risk of aGVHD III–IV was lower with BM than with PB + ATG and the risk of TRM was similar between the groups. The CI of relapse at two years was 28%, 25% and 30% with BM, PB and PB + ATG, respectively (*p* = 0.31), and there was no difference after weighting. The probability of two-year overall survival (OS) was 60.3%, 54.1% and 42.4% with BM, PB and PB + ATG, respectively (*p* < 0.05), but there was no difference after adjustments. Then, after adjustments, the risk of two-year GVHD-and-relapse-free-survival (GRFS) remained lower with BM than with PB + ATG (Additional file 3: Table S3).

**Fig. 1:**
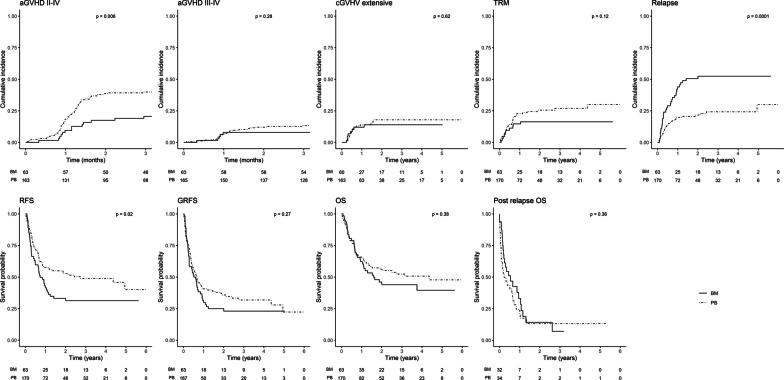
Cumulative incidence of outcomes for AL-MDS-MPS with NMA conditioning, according to graft

Analyses then focused on a subgroup of diseases at high risk of relapse, AL-MDS-MPS, and excluding patients receiving ATG. Conditioning regimens were grouped into two categories: NMA Baltimore-type and more intensive regimens (MAC and reduced intensity conditioning (RIC) excluding NMA). In the setting of NMA regimen and after adjustments, there was a twofold increased risk of aGVHD II–IV as well as a twofold lower risk of relapse with PB than with BM grafts (Fig. 1; Additional file 3: Table S4).

**Fig. 2:**
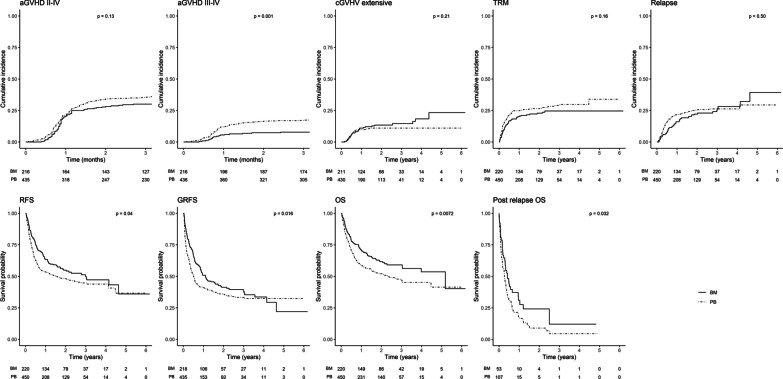
Cumulative incidence of outcomes for AL-MDS-MPS with intensive conditioning, according to graft

With more intensive conditioning regimens, the risk of aGVHD III–IV remained twice higher with PB without any difference in risk of others outcomes of interests, in particular without impact on relapse (Fig. 2; Additional file 3: Table S5).

## Discussion

In line with previous studies reporting aGVHD rates of 25% for transplants with BM grafts versus 40% with PB grafts, we found that PB graft was associated with higher rates of aGVHD compared to BM [4–10]. Adding ATG did not decrease the rate of GVHD. This is in contradiction with previous studies reporting a decrease in the rate of cGVHD when ATG was added to PTCy [11]. However, these studies were based on a limited number of patients, had included various doses of ATG or Cy-PT, and reported various grades of cGVHD. We report that in the subgroup of patients with AL-MDS-MPS, the risk of relapse is greatly increased with BM associated with NMA, whereas this risk is no longer observed when conditioning is intensified. Other studies have reported an increased risk of relapse in patients with leukemia after BM graft, or a reduction in relapses after MAC [5, 12]. However, no study has performed a combined analysis of relapse risk in leukemia patients according to graft type and conditioning intensity, possibly due to insufficient population size. This is the largest series of haploidentical transplants with PTCy reported to date. Although retrospective, statistical analysis using the propensity score makes it possible to homogenize the sub-populations and make them comparable. The large number of patients studied makes it possible to analyze the impact of the “intensity of conditioning regimen” factor in addition to graft type and disease type. We feel it is important to remember that graft outcome depends on many factors, and that these three elements must be considered to optimize the hematological treatment (Additional file 2).

### Supplementary Information

**Additional file 1.** Supplementary materials and methods.

**Additional file 2.** Supplementary discussion.

**Additional file 3.** Supplementary tables.

**Additional file 4.** Supplementary figures.

## Data Availability

All clinical and biological data have been shared within the Société Francophone de Greffe de Moelle et de Thérapie Cellulaire (SFGM-TC) Promise database. External users with a formal analysis plan may request access to these data from corresponding author.

## Abbreviations

ATG: Anti-thymoglobulin
BM: Bone marrow
CI: Cumulative incidence
GVHD: Graft-versus-host disease
aGVHD: Acute graft-versus-host disease
cGVHD: Chronic graft-versus-host disease
GRFS: GVHD-free/relapse-free survival
HR: Hazard ratio
IQR: Interquartile range
MAC: Myeloablative conditioning
NMA: Non-myeloablative
OS: Overall survival
PB: Peripheral blood
PTCy: Post-transplant cyclophosphamide
RIC: Reduced intensity conditioning
TRM: Treatment-related mortality
