# Supplementary material for: Bone marrow graft versus peripheral blood graft in haploidentical hematopoietic stem cells transplantation: a retrospective analysis in1344 patients of SFGM-TC registry
Source: J Hematol Oncol. 2024 Jan 7;17:2. doi: 10.1186/s13045-023-01515-4 (PMC10773006; doi:10.1186/s13045-023-01515-4)
Supplement: Supplementary file 1 — Additional file 1. Supplementary materials and methods. [file 13045_2023_1515_MOESM1_ESM.docx]

**Additional file 1: Supplementary materials and methods**

**Study design and patients**

In this retrospective registry-based analysis, eligible patients were at least 18 years old and received T-cell repleted graft from a haploidentical familial donor between May 2012 and December 2019 in 37 French centers. They were treated for acute myeloid or lymphoblastic leukemia (AML or ALL), myelodysplastic or myeloproliferative syndrome, chronic lymphocytic leukemia, non-Hodgkin's or Hodgkin’s lymphoma or multiple myeloma. MAC, RIC, and NMA Baltimore-type conditioning regimen were defined according to previous descriptions (1-3). The disease risk index (DRI) considers disease type, disease status at transplant, cytogenetic risk for leukemia and myelodysplastic syndrome on the one hand and disease type and disease status at transplant for lymphoma on the other hand (4). High-dose PTCy (50mg/Kg for two days) combined with anticalcineurin and mycophenolate mofetil (MMF) were administered for GVHD prophylaxis. ATG has been added in some patients who received PB graft. The Institutional Review Board of the SFGM-TC approved the study. All patients provided written informed consent for their data to be used for clinical research in accordance with the modified Declaration of Helsinki and Good Clinical Practice guidelines.

**Statistical analysis and endpoints**

Patients’ characteristics are described using median and interquartile range (IQR) for quantitative variables and count and percentages for qualitative variables. Characteristics were compared between three graft sources using Kruskall Wallis test or Chi-Squared test. Overall survival (OS) was defined as time from transplantation to death; GVHD-free/relapse-free survival (GRFS) as time from transplantation to either malignancy relapse, grade III-IV GVHD, extensive chronic GVHD or death (5, 6). Relapse was defined as disease recurrence (morphologic, cytogenetic or molecular) after a period of complete remission, considering death without relapse as competing event. Time to GVHD was defined as time between transplantation and either grade II-IV aGVHD, grade III-IV aGVHD or extensive chronic GVHD, considering death without GVHD as competing event. Acute and chronic GVHD were assessed according to modified Seattle Glucksberg criteria (7) and revised Seattle criteria (8) respectively. Toxicities were determined according to Common Terminology Criteria for Adverse Events of the National Cancer Institute. Treatment related mortality (TRM) was defined as time between transplantation and transplantation-related death without previous relapse. Time to platelet recovery was defined as time between transplantation and platelet count above 20 G/L without transfusion during the previous 7 days, in the subset of patients with thrombocytopenia and considering death without platelet recovery and absence of platelet recovery after 100 days as competing endpoints. Time to neutrophil recovery was defined as time between transplantation and neutrophil count above 500/mm^3^ during three consecutive days, in the subset of patients with neutropenia and considering death without neutrophil recovery and absence of neutrophil recovery after 100 days as competing endpoints. When comparing graft sources, propensity score weighting analysis using overlap weights was run. In this analysis, each patient is weighted according to his predicted probability of having received the treatment of the opposite group, estimated by the propensity score (PS). For each pairwise comparison in each population (total population or AL and Myeloid Diseases with NMA Conditioning population) PS was estimated using logistic regression and patients are weighted using either PS for control group or (1-PS) for experimental group. The resulting pseudo-populations are exactly balanced for each covariate included in the PS and mimic the characteristics of a pragmatic randomized trial that is highly inclusive, excluding no study participants from the available sample but emphasizing the comparison of patients at clinical equipoise (tables S6 S7 and S8, and figures S1 and S2). Effective sample size of pseudo-populations is presented to illustrate the loss of precision related to weighting. Covariate included in the PS model were factors with prognostic effect on the endpoints: for the three pairwise comparisons between graft sources, recipient’s age, DRI score, ABO mismatch, sex mismatch, donor sex, recipient CMV status, conditioning regimen were included; for subgroup analyses in specific conditioning regimen, conditioning variable was not included. Comparison between graft sources on endpoints were performed using weighted Cox proportional hazard models or weighted Cause-specific Cox proportional hazard models. All statistical analyses were performed using R software. A p-value below 0.05 was considered as significant.

**References**

1. Luznik L, O’Donnell PV, Symons HJ, Chen AR, Leffell MS, Zahurak M, et al. HLA-haploidentical bone marrow transplantation for hematologic malignancies using nonmyeloablative conditioning and high-dose, posttransplantation cyclophosphamide. Biol Blood Marrow Transplant J Am Soc Blood Marrow Transplant. juin 2008;14(6):641‑50.
2. Brunstein CG, Fuchs EJ, Carter SL, Karanes C, Costa LJ, Wu J, et al. Alternative donor transplantation after reduced intensity conditioning: results of parallel phase 2 trials using partially HLA-mismatched related bone marrow or unrelated double umbilical cord blood grafts. Blood. 14 juill 2011;118(2):282‑8.
3. Bacigalupo A, Dominietto A, Ghiso A, Di Grazia C, Lamparelli T, Gualandi F, et al. Unmanipulated haploidentical bone marrow transplantation and post-transplant cyclophosphamide for hematologic malignanices following a myeloablative conditioning: an update. Bone Marrow Transplant. juin 2015;50 Suppl 2:S37-39.
4. Armand P, Gibson CJ, Cutler C, Ho VT, Koreth J, Alyea EP, et al. A disease risk index for patients undergoing allogeneic stem cell transplantation. Blood. 26 juill 2012;120(4):905‑13.
5. Holtan SG, DeFor TE, Lazaryan A, Bejanyan N, Arora M, Brunstein CG, et al. Composite end point of graft-versus-host disease-free, relapse-free survival after allogeneic hematopoietic cell transplantation. Blood. 19 févr 2015;125(8):1333‑8.
6. Aversa F, Tabilio A, Velardi A, Cunningham I, Terenzi A, Falzetti F, et al. Treatment of high-risk acute leukemia with T-cell-depleted stem cells from related donors with one fully mismatched HLA haplotype. N Engl J Med. 22 oct 1998;339(17):1186‑93.
7. Glucksberg H, Storb R, Fefer A, Buckner CD, Neiman PE, Clift RA, et al. Clinical manifestations of graft-versus-host disease in human recipients of marrow from HL-A-matched sibling donors. Transplantation. oct 1974;18(4):295‑304.
8. Filipovich AH, Weisdorf D, Pavletic S, Socie G, Wingard JR, Lee SJ, et al. National Institutes of Health consensus development project on criteria for clinical trials in chronic graft-versus-host disease: I. Diagnosis and staging working group report. Biol Blood Marrow Transplant J Am Soc Blood Marrow Transplant. déc 2005;11(12):945‑56.
