# Supplementary material for: Bone marrow graft versus peripheral blood graft in haploidentical hematopoietic stem cells transplantation: a retrospective analysis in1344 patients of SFGM-TC registry
Source: J Hematol Oncol. 2024 Jan 7;17:2. doi: 10.1186/s13045-023-01515-4 (PMC10773006; doi:10.1186/s13045-023-01515-4)
Supplement: Supplementary file 2 — Additional file 2. Supplementary discussion. [file 13045_2023_1515_MOESM2_ESM.docx]

**Additional file 2: Supplementary discussion**

The main conclusions of our study are: (i)PB use increases the risk of aGVHD compared to BM in the overall population. (ii)Adding ATG to PB does not decrease the risk of GVHD. (iii) In the subgroup of patients with acute leukemia and MDS/MPS, the risk of relapse is greatly increased with a bone marrow graft associated with non-myeloablative conditioning. This risk is no longer observed when conditioning regimens are intensified.

The increased risk of GVH when using a PB graft has prompted a number of teams to add ATG to PTCy. In our study, analyses performed on the overall population have shown that the combination of ATG and PTCy for GVHD prophylaxis when a PB graft is administered does not decrease the rates of acute or chronic GVHD. On the contrary, it appears that the addition of ATG during PB transplantation remains associated with an increased incidence of severe acute GVHD and TRM (probably related to high rates of severe infections), compared with BM transplants, without a parallel benefit on relapse rates. This is in contradiction with previous studies reporting a decrease in the rate of GVHD when ATG was added to PTCy (1). Five studies have compared PTCy + ATG vs. PTCy alone. Two studies found a significant difference in the incidence of acute GVHD. Unlike our study, most of these studies found a significant difference in the incidence of chronic GVHD, with cGVHD (or ext/severe cGVHD) rates ranging from 0 to 24% with the addition of ATG to PTCy. In our study, patients treated with PB+ATG differ in many ways from patients treated with PB without ATG. In terms of DRI score, patients in the PB+ATG group had higher-risk pathologies than patients treated with PB alone (30% high risk and 7% very high risk in the PB+ATG group vs. 20% high risk and 2% very high risk in the PB group). Moreover, 93% of patients treated in the PB+ATG group received intensive conditioning (and therefore 7% Baltimore-type conditioning) vs. 64% in the PB alone group (i.e. 36% Baltimore-type conditioning). This much higher rate of high-risk relapse pathologies, combined with a higher rate of more toxic conditioning, may contribute to a higher incidence of GVHD, TRM and worse GRFS. However, after statistical weighting, the results were unchanged and we found no benefit of ATG on either acute or chronic extensive GVH. Previous published studies involved a limited number of patients (n=21 to 69 for PTCy+ATG cohorts) with different doses of PTCy and ATG. In our retrospective registry study, ATG doses were also variable (ranging from 2.5mg/Kg to 10mg/Kg total dose ATG) but with no information on the type of ATG used or the precise method of administration. In addition, when PTCy was administered, doses varied from 50mg/Kg to 100mg/Kg total dose). Finally, our data concerned the incidence of extensive GVHD, whereas the other studies focused on chronic GVH in general or severe/moderate cGVHD. It is possible that the variability of administration of ATG/PTCy and the small number of patients in previously published studies explain these contradictory results.

In studies of MUD or MRD transplants, engraftment or hematological recovery was faster with PB graft than with BM graft, probably correlated with enrichment of CD34^+^ cells. However, in our study and in accordance with previous reports, this benefit of PB graft in terms of engraftment or platelet recovery was not found when the overall population of haploidentical transplants was analyzed. Certainly, within the subgroup of myeloid malignancies and after Baltimore-type NMA conditioning, platelet recovery seems to be faster and more important with a PB graft than with a BM graft. However, it is interesting to note that when more intensive conditioning is used, this platelet recovery seems to be slower with PB graft than with BM graft. It is indeed possible that the increase in the rate of acute GVHD with PB graft has a negative impact on platelet recovery, on one hand because of the GVHD itself and on the other hand due to associated drugs and infections.

Regarding relapse, we report that the risk of relapse is doubled in the acute leukemia/MDS/MPS group when patients are transplanted with BM and a NMA conditioning regimen. This risk is no longer observed after more intensified regimens (MAC, Reduced toxicity (RTC) or reduced intensity (RIC) conditioning). Two retrospective studies noted an increased risk of relapse in patients treated for acute leukemia receiving a BM transplant: a study including 86 patients (2) and a registry study with a larger enrollment (671 patients) but not distinguishing between RIC and MAC regimens (3).

A key element of our work was to question the concept of conditioning intensity. Indeed, PTCy intensifies conditioning toxicity in a nontrivial way. The reduced-dose TBF regimen associated with PTCy is more akin to reduced toxicity conditioning (RTC) than to reduced intensity conditioning (4-7). Thus, the usual RIC/MAC distinction did not make sense in the setting of haploidentical transplants with PTCy; NMA Baltimore-type conditioning was therefore distinguished from the more intensive conditionings (including other RIC, RTC and MAC), a distinction that seemed to be clinically more relevant. Bashey et al compared haploidentical transplantation BM (n = 481) or PB (n = 190) and reported that the higher relapse risks after transplantation of BM were limited to patients with leukemia (HR, 1.73; P = .002) and not lymphoma (HR, 0.87; P =.64), irrespective of conditioning intensity. However, the vast majority of patients transplanted with bone marrow had non-myeloablative conditioning (82%). The analysis was not performed on the sub-population with more intensive conditioning, probably due to lack of numbers. In our study, the majority of BM patients had MAC or RTC conditioning, allowing us to perform analyze on the subgroup of patients treated for acute leukemia or myeloid malignancy with intensive conditioning and bone marrow. In the meta-analysis by Arcuri et al, the use of marrow was associated with an increased risk of relapse, and the use of MAC was associated with a reduction in relapses (8). This does not contradict our study. Again, there was no analysis of the use of BM with MAC vs. BM with NMA regimen.

There are two other platforms for haploidentical transplants: the TCD approach and the Beijing’s approach. The TCD platform involves combining high doses of ATG with depletion of the graft's T lymphocytes via positive selection of CD34+ stem cells. Since T-cells are extensively depleted from the graft in vitro, it seems difficult to apply PTCy which acts mainly by eliminating alloreactive T-cells that are activated after graft infusion. Beijing's approach based on high doses of ATG is very interesting and gives excellent results. However, it seems difficult to project these conclusions to the Beijing approach, which combines G-CSF primed bone marrow and peripheral blood, as well as G-CSF primed DLI after transplant. However, it should be noted that a recent study compared a reduced-dose PTCy/ATG to a standard-dose ATG group ('Beijing Protocol', ATG: 10 mg/kg) in a multicenter controlled randomized study (9). The addition of low-dose ATG to reduced-dose PTCy provided promising results and merits to be further investigated.

**References**

1. Duléry R, Brissot E, Mohty M. Combining post-transplant cyclophosphamide with antithymocyte globulin for graft-versus-host disease prophylaxis in hematological malignancies. Blood Rev. 2023 Apr 11:101080. doi: 10.1016/j.blre.2023.101080. Epub ahead of print. PMID: 37085459.
2. O’Donnell PV, Eapen M, Horowitz MM, Logan BR, DiGilio A, Brunstein C, et al. Comparable outcomes with marrow or peripheral blood as stem cell sources for hematopoietic cell transplantation from haploidentical donors after non-ablative conditioning: a matched-pair analysis. Bone Marrow Transplant. déc 2016;51(12):1599‑601
3. Bashey A, Zhang MJ, McCurdy SR, St. Martin A, Argall T, Anasetti C, et al. Mobilized Peripheral Blood Stem Cells Versus Unstimulated Bone Marrow As a Graft Source for T-Cell–Replete Haploidentical Donor Transplantation Using Post-Transplant Cyclophosphamide. J Clin Oncol. 10 sept 2017;35(26):3002-9.
4. Sanz J, Boluda JCH, Martín C, González M, Ferrá C, Serrano D, et al. Single-unit umbilical cord blood transplantation from unrelated donors in patients with hematological malignancy using busulfan, thiotepa, fludarabine and ATG as myeloablative conditioning regimen. Bone Marrow Transplant. oct 2012;47(10):1287‑93
5. Giannotti F, Labopin M, Shouval R, Sanz J, Arcese W, Angelucci E, et al. Haploidentical transplantation is associated with better overall survival when compared to single cord blood transplantation: an EBMT-Eurocord study of acute leukemia patients conditioned with thiotepa, busulfan, and fludarabine. J Hematol OncolJ Hematol Oncol. 30 août 2018;11(1):110.
6. Duléry R, Bastos J, Paviglianiti A, Malard F, Brissot E, Battipaglia G, et al. Thiotepa, Busulfan, and Fludarabine Conditioning Regimen in T Cell-Replete HLA-Haploidentical Hematopoietic Stem Cell Transplantation. Biol Blood Marrow Transplant J Am Soc Blood Marrow Transplant. juill 2019;25(7):1407‑15.
7. Bregante S, Dominietto A, Ghiso A, Raiola AM, Gualandi F, Varaldo R, et al. Improved Outcome of Alternative Donor Transplantations in Patients with Myelofibrosis: From Unrelated to Haploidentical Family Donors. Biol Blood Marrow Transplant J Am Soc Blood Marrow Transplant. févr 2016;22(2):324‑9.
8. Arcuri LJ, Hamerschlak N, Rocha V, Bonfim C, Kerbauy MN. Outcomes after Haploidentical Hematopoietic Cell Transplantation with Post-Transplantation Cyclophosphamide: A Systematic Review and Meta-Analysis Comparing Myeloablative with Reduced-Intensity Conditioning Regimens and Bone Marrow with Peripheral Blood Stem Cell Grafts. Transplant Cell Ther. 2021 Sep;27(9):782.e1-782.e7. doi: 10.1016/j.jtct.2021.06.011. Epub 2021 Jun 16. PMID: 34146733.
9. Zhang W, Gui R, Zu Y, Zhang B, Li Z, Zhang Y, Wang X, Guo S, Zhan X, Fu Y, Song Y, Zhou J. Reduced-dose post-transplant cyclophosphamide plus low-dose post-transplant anti-thymocyte globulin as graft-versus-host disease prophylaxis with fludarabine-busulfan-cytarabine conditioning inhaploidentical peripheral blood stem cell transplantation: A multicentre, randomized controlled clinical trial. Br J Haematol. 2023 Jan;200(2):210-221. doi: 10.1111/bjh.18483. Epub 2022 Oct 6. PMID: 36200642.
