# Supplementary material for: Bone marrow graft versus peripheral blood graft in haploidentical hematopoietic stem cells transplantation: a retrospective analysis in1344 patients of SFGM-TC registry
Source: J Hematol Oncol. 2024 Jan 7;17:2. doi: 10.1186/s13045-023-01515-4 (PMC10773006; doi:10.1186/s13045-023-01515-4)
Supplement: Supplementary file 3 — Additional file 3. Supplementary tables. [file 13045_2023_1515_MOESM3_ESM.docx]

**Additional file 3: Supplementary tables**

**Table S1: Patient, Disease and Transplant Characteristics**

|  |  | **N** | **BM** | **N** | **PB** | **N** | **PB+ATG** | **p-value** |  |
| --- | --- | --- | --- | --- | --- | --- | --- | --- | --- |
|  |  | 371 | % | 776 | % | 197 | % |  |  |
| **Age at treatment**  (Median age, IQR)  (mean(SD)) | Years | 371 | 51 [33.34;61.73]  47.74 (16.09) | 776 | 57.91 [43.39;65.02]  53.33 (14.75) | 197 | 55.81 [42.39;62.6]  52.13 (13.64) | <0.0001 |  |
| **Sexe** | F | 144 | 38.81 % | 285 | 36.73 % | 77 | 39.09 % | 0.72 |  |
|  | M | 227 | 61.19 % | 491 | 63.27 % | 120 | 60.91 % |  |  |
| **Hematological disease group** | AL* | 200 | 53.91 % | 439 | 56.57 % | 112 | 56.85 % | 0.028 |  |
|  | Lymphoid | 88 | 23.72 % | 156 | 20.1 % | 26 | 13.2 % |  |  |
|  | Myeloid | 83 | 22.37 % | 181 | 23.32 % | 59 | 29.95 % |  |  |
| **Disease Risk Index** | Low | 76 | 22.29 % | 148 | 20.5 % | 22 | 12.36 % | <0.0001 |  |
|  | Intermediate | 195 | 57.18 % | 416 | 57.62 % | 91 | 51.12 % |  |  |
|  | High | 65 | 19.06 % | 142 | 19.67 % | 53 | 29.78 % |  |  |
|  | Very high | 5 | 1.47 % | 16 | 2.22 % | 12 | 6.74 % |  |  |
| **Conditioning regimen** | NMA-Baltimore | 122 | 32.88 % | 276 | 35.71 % | 14 | 7.11 % | <0.0001 |  |
|  | MAC | 152 | 40.97 % | 187 | 24.19 % | 70 | 35.53 % |  |  |
|  | RIC | 97 | 26.15 % | 310 | 40.1 % | 113 | 57.36 % |  |  |
| **CMV status recipient** | Negative | 145 | 39.73 % | 296 | 38.44 % | 80 | 41.24 % | 0.75 |  |
|  | Positive | 220 | 60.27 % | 474 | 61.56 % | 114 | 58.76 % |  |  |
| **Number of infused CD34^+^ cells**  (median, IQR)  (mean(SD)) | 10^6^/kg | 339 | 2.62  [1.81 ;3.74]  3.03 (2.15) | 733 | 6.33  [4.85 ;8.22]  6.78 (2.87) | 189 | 5.99  [5 ; 7.6]  6.40 (2.36) | <0.0001 |  |
| **Number of infused CD3^+^ cells**  (median, IQR)  (mean(SD)) | 10^6^/kg | 286 | 27  [19.05 ;34.62]  37.74 (57.25) | 687 | 269  [193 ;362]  297.2 (166) | 159 | 250  [170 ;349]  257.9 (127.9) | <0.0001 |  |

***Abbreviations:*** *BM: bone marrow; PB: peripheral blood stem cells; ATG: anti-thymoglobulin; AL: acute leukemia; RIC: reduced intensity conditioning; MAC: myeloablative conditioning; NMA: non myeloablative conditioning; CMV: cytomegalovirus.*

******* *”AL” include acute lymphoblastic leukemia, acute myeloblastic leukemia, biphenotypic acute leukemia, undifferentiated acute leukemia, “myeloid diseases” include myelodysplastic syndrome and myeloproliferative syndrome and "lymphoid diseases" include Hodgkin's lymphoma, non-Hodgkin's lymphoma, chronic lymphocytic leukemia and multiple myeloma.*

**Table S2: Transplant Engraftment**

|  | **BM** | **PB** | **PB+ATG** |
| --- | --- | --- | --- |
| **Median time to platelet recovery [95%CI] (days)** | 26 [25-27] | 27 [26-29] | 26 [23-30] |
| **Median time to neutrophil recovery [95%CI] (days)** | 20 [20-21] | 20 [19-20] | 20 [19-21] |
| **Cumulative incidence of neutrophil recovery at 1 month** | 90% [86.4-92.7] | 89% [86.5-91.1] | 89.5% [84.2-93.2] |

***Abbreviations:*** *BM: bone marrow; PB: peripheral blood stem cells; ATG: anti-thymoglobulin; CI: confidence interval*

**Table S3: Effect of Graft Type on Transplant Outcomes in Overall Population – Pairwise comparisons**

|  | **Hazard ratio (95% CI, p)**  **BM vs. PB**  *PB=1* | **Hazard ratio (95% CI, p)**  **PB vs. PB+ATG**  *PB+ATG=1* | **Hazard ratio (95% CI, p)**  **BM vs. PB+ATG**  *PB+ATG =1* |
| --- | --- | --- | --- |
| **aGVHD II-IV** | 0.65 (0.52-0.83, p=0.0004) | 1.31 (0.96-1.77, p=0.084) | 0.98 (0.68-1.4, p=0.91) |
| **aGVHD III-IV** | 0.49 (0.32-0.74, p=0.0007) | 1.05 (0.69-1.61, p=0.81) | 0.48 (0.27-0.85, p=0.012) |
| **Extensive cGVHD** | 0.98 (0.66-1.45, p=0.92) | 1.3 (0.73-2.29, p=0.37) | 1.2 (0.64-2.24, p=0.57) |
| **TRM** | 0.84 (0.63-1.11, p=0.22) | 0.91 (0.66-1.25, p=0.54) | 0.72 (0.48-1.07, p=0.10) |
| **Relapse** | 1.13 (0.87-1.47, p=0.35) | 0.96 (0.69-1.33, p=0.80) | 0.85 (0.58-1.25, p=0.41) |
| **GRFS (GVHD or relapse event)** | 0.9 (0.76-1.07, p=0.24) | 0.94 (0.76-1.17, p=0.59) | 0.75 (0.58-0.97, p=0.029) |
| **OS (death event)** | 0.88 (0.72-1.09, p=0.24) | 0.93 (0.73-1.19, p=0.57) | 0.76 (0.56-1.02, p=0.068) |

***Abbreviations:*** *GVHD: Graft versus Host Disease; aGVHD: three-month acute GVHD; two-years cGVHD: chronic GVHD; TRM: two-years toxicities related mortality; GRFS; two-years GVHD- and relapse-free survival; OS: two-years overall survival; BM: bone marrow; PB: peripheral blood stem cells; ATG: anti-thymoglobulin; CI: confidence interval*

***NB:*** *Hazard Ratio are reported after weighting with propensity score.*

**Table S4: Effect of Graft Type on Transplant Outcomes for AL- MDS-MPS with NMA Conditioning**

| **Outcomes** | **Cumulative incidence** | **Hazard Ratio**  **(95% CI)** | **p** |
| --- | --- | --- | --- |
| **Grade 2-4 aGVHD**  *BM*  *PB* | 20.6%  39.9% | 1  2.24 (1.2-4.19) | 0.01 |
| **Grade 3-4 aGVHD**  *BM*  *PB* | 7.9%  13.3% | 1  1.93 (0.68-5.48) | 0.21 |
| **Extensive cGVHD**  *BM*  *PB* | 14.0%  17.8% | 1  1.13 (0.48-2.64) | 0.78 |
| **TRM**  *BM*  *PB* | 16.3%  25.5% | 1  1.57 (0.75-3.29) | 0.23 |
| **Relapse**  *BM*  *PB* | 50.5%  21.7% | 1  0.50 (0.3-0.85) | 0.01 |
| **GRFS**  *BM*  *PB* | 25.0%  35.8% | 1  0.89 (0.61-1.3) | 0.55 |
| **OS**  *BM*  *PB* | 44.1%  56.2% | 1  0.95 (0.61-1.48) | 0.83 |
| **RFS**  *BM*  *PB* | 33.2%  52.8% | 1  0.74 (0.49-1.12) | 0.15 |

***Abbreviations:*** *AL: acute leukemia; MDS : myelodysplastic syndrome; MPS: myeloproliferative syndrome;* *GVHD: Graft versus Host Disease; aGVHD: three-month acute GVHD; two-years cGVHD: chronic GVHD; TRM: two-years toxicities related mortality; GRFS; two-years GVHD- and relapse-free survival; OS: two-years overall survival; RFS: two-years relapse-free survival; BM: bone marrow; PB: peripheral blood stem cells; CI: confidence interval*

***NB:*** *Cumulative incidences are calculated without weighting. Hazard Ratio are reported after weighting with propensity score. For GRFS, hazard ratio reports the risk of GVHD or relapse event; for OS and RFS, hazard ratio reports the risk of death event.*

**Table S5: Effect of Graft Type on Transplant Outcomes for AL-MDS-MPS with Intensive Conditioning**

| **Outcomes** | **Cumulative incidence** | **Hazard Ratio**  **(95% CI)** | **p** |
| --- | --- | --- | --- |
| **Grade 2-4 aGVHD**  *BM*  *PB* | 30.1%  35.4% | 1  1.17 (0.86-1.58) | 0.32 |
| **Grade 3-4 aGVHD**  *BM*  *PB* | 7.9%  17.2% | 1  2.0 (1.17-3.43) | 0.01 |
| **Extensive cGVHD**  *BM*  *PB* | 13.4%  11.0% | 1  0.85 (0.52-1.4) | 0.52 |
| **TRM**  *BM*  *PB* | 22.9%  26.5% | 1  1.25 (0.87-1.81) | 0.23 |
| **Relapse**  *BM*  *PB* | 22.2%  25.3% | 1  1.06 (0.74-1.51) | 0.75 |
| **GRFS**  *BM*  *PB* | 41.4%  35.8% | 1  1.19 (0.94-1.49) | 0.14 |
| **OS**  *BM*  *PB* | 61.6%  52% | 1  1.23 (0.94-1.63) | 0.14 |
| **RFS**  *BM*  *PB* | 54.9%  48.1% | 1  1.15 (0.89-1.48) | 0.28 |

***Abbreviations:*** *AL: acute leukemia; MDS : myelodysplastic syndrome; MPS: myeloproliferative syndrome;* *GVHD: Graft versus Host Disease; aGVHD: three-month acute GVHD; two-years cGVHD: chronic GVHD; TRM: two-years toxicities related mortality; GRFS; two-years GVHD- and relapse-free survival; OS: two-years overall survival; RFS: two-years relapse-free survival; BM: bone marrow; PB: peripheral blood stem cells; CI: confidence interval*

***NB:*** *Cumulative incidences are calculated without weighting. Hazard Ratio are reported after weighting with propensity score. For GRFS, hazard ratio reports the risk of GVHD or relapse event; for OS and RFS, hazard ratio reports the risk of death event.*

**Table S6: Weighted populations for pairwise comparisons in the global population**

|  | **BM vs PB** | | | **BM vs PB+ATG** | | | **PB vs PB+ATG** | | |
| --- | --- | --- | --- | --- | --- | --- | --- | --- | --- |
|  | PB | BM | SMD | PB+ATG | BM | SMD | PB+ATG | PB | SMD |
| **N** (ESS) | 619.7 | 326.2 |  | 156.6 | 230.2 |  | 172.5 | 500.7 |  |
| **Age at treatment** (mean(SD)) | 49.22 (15.67) | 49.22 (15.52) | <0.001 | 50.43 (14.21) | 50.43 (14.80) | <0.001 | 52.13 (13.62) | 52.13 (14.57) | <0.001 |
| **Number of infused CD34+ cells** (mean (SD)) | 6.78 (2.86) | 2.94 (1.91) | 1.579 | 6.39 (2.34) | 2.98 (1.81) | 1.628 | 6.41 (2.38) | 7.00 (3.06) | 0.215 |
| **Number of infused CD3+ cells** (mean (SD)) | 293.85 (169.96) | 36.11 (53.27) | 2.046 | 259.22 (127.42) | 38.09 (56.12) | 2.246 | 261.09 (128.44) | 295.40 (169.80) | 0.228 |
| **Sexe** = Male (%) | 399.7 (64.5) | 200.9 (61.6) | 0.062 | 92.2 (58.9) | 141.6 (61.5) | 0.055 | 102.1 (59.2) | 311.9 (62.3) | 0.063 |
| **Hematological disease group** (%) |  |  | 0.119 |  |  | 0.049 |  |  | 0.122 |
| AL | 359.4 (58.0) | 169.6 (52.0) |  | 89.4 (57.1) | 127.5 (55.4) |  | 95.6 (55.4) | 307.4 (61.4) |  |
| Lymphoid | 141.3 (22.8) | 83.8 (25.7) |  | 24.3 (15.5) | 39.8 (17.3) |  | 26.2 (15.2) | 63.6 (12.7) |  |
| Myeloid | 119 (19.2) | 72.4 (22.2) |  | 43.1 (27.5) | 62.8 (27.3) |  | 50.7 (29.4) | 129.7 (25.9) |  |
| **Disease Risk Index** (%) |  |  | <0.001 |  |  | <0.001 |  |  | <0.001 |
| low | 131.4 (21.2) | 69.2 (21.2) |  | 23.8 (15.2) | 35 (15.2) |  | 24.1 (14.0) | 70.1 (14.0) |  |
| int | 356.3 (57.5) | 187.6 (57.5) |  | 85.8 (54.8) | 126.1 (54.8) |  | 91.9 (53.3) | 266.9 (53.3) |  |
| high | 122.1 (19.7) | 64.3 (19.7) |  | 43.1 (27.5) | 63.3 (27.5) |  | 49 (28.4) | 142.2 (28.4) |  |
| very high | 9.3 ( 1.5) | 4.9 ( 1.5) |  | 3.9 ( 2.5) | 5.8 ( 2.5) |  | 7.4 ( 4.3) | 21.5 ( 4.3) |  |
| **Conditioning regimen** (%) |  |  | <0.001 |  |  | <0.001 |  |  | <0.001 |
| NMA-Baltimore | 220 (35.5) | 115.8 (35.5) |  | 16.6 (10.6) | 24.4 (10.6) |  | 15.2 ( 8.8) | 44.1 ( 8.8) |  |
| MAC | 214.4 (34.6) | 112.9 (34.6) |  | 67.2 (42.9) | 98.8 (42.9) |  | 61.2 (35.5) | 177.7 (35.5) |  |
| RIC | 185.3 (29.9) | 97.5 (29.9) |  | 72.8 (46.5) | 107 (46.5) |  | 96.1 (55.7) | 278.9 (55.7) |  |
| **CMV status recipient** = Positive (%) | 370 (59.7) | 194.7 (59.7) | <0.001 | 92.2 (58.9) | 135.6 (58.9) | <0.001 | 102.1 (59.2) | 296.4 (59.2) | <0.001 |

***Abbreviations:*** *SMD: Standardized Mean Difference;* *BM: bone marrow; PB: peripheral blood stem cells; ATG: anti-thymoglobulin; AL: acute leukemia; RIC: reduced intensity conditioning; MAC: myeloablative conditioning; NMA: non myeloablative conditioning; CMV: cytomegalovirus.*

******* *”AL” include acute lymphoblastic leukemia, acute myeloblastic leukemia, biphenotypic acute leukemia, undifferentiated acute leukemia, “myeloid diseases” include myelodysplastic syndrome and myeloproliferative syndrome and "lymphoid diseases" include Hodgkin's lymphoma, non-Hodgkin's lymphoma, chronic lymphocytic leukemia and multiple myeloma.*

**Table S7: Weighted populations for pairwise comparisons in the subgroup of patients with AL-MDS-MPS with NMA conditioning**

|  | **BM** | **PB** | **SMD** |
| --- | --- | --- | --- |
| **N** (ESS) | 55.2 | 130.2 |  |
| **Age at treatment** (mean(SD)) | 57.02 (13.02) | 57.02 (14.44) | <0.001 |
| **Number of infused CD34+ cells** (mean (SD)) | 2.69 (0.94) | 6.86 (2.42) | 2.276 |
| **Number of infused CD3+ cells** (mean (SD)) | 27.47 (12.98) | 291.25 (121.43) | 3.055 |
| **Sexe** = Male (%) | 36.9 (66.8) | 81.2 (62.4) | 0.093 |
| **Hematological disease group** (%) |  |  | 0.075 |
| AL | 37.1 (67.2) | 92.1 (70.7) |  |
| Lymphoid | 0 ( 0.0) | 0 ( 0.0) |  |
| Myeloid | 18.1 (32.8) | 38.1 (29.3) |  |
| **Disease Risk Index** (%) |  |  | <0.001 |
| low | 11.3 (20.4) | 26.6 (20.4) |  |
| int | 30.4 (55.1) | 71.7 (55.1) |  |
| high | 11.9 (21.6) | 28.1 (21.6) |  |
| very high | 1.6 ( 2.9) | 3.8 ( 2.9) |  |
| **CMV status** **recipient** = Positive (%) | 38.4 (69.6) | 90.6 (69.6) | <0.001 |

***Abbreviations:*** *SMD: Standardized Mean Difference;* *BM: bone marrow; PB: peripheral blood stem cells; ATG: anti-thymoglobulin; AL: acute leukemia; MDS: myelodysplastic syndrome; MPS: myeloproliferative syndrome; RIC: reduced intensity conditioning; MAC: myeloablative conditioning; NMA: non myeloablative conditioning; CMV: cytomegalovirus.*

******* *”AL” include acute lymphoblastic leukemia, acute myeloblastic leukemia, biphenotypic acute leukemia, undifferentiated acute leukemia, “myeloid diseases” include myelodysplastic syndrome and myeloproliferative syndrome and "lymphoid diseases" include Hodgkin's lymphoma, non-Hodgkin's lymphoma, chronic lymphocytic leukemia and multiple myeloma.*

**Table S8: Weighted populations for pairwise comparisons in the subgroup of patients with AL- MDS-MPS with intensive conditioning**

|  | **BM** | **PB** | **SMD** |
| --- | --- | --- | --- |
| **N** (ESS) | 189 | 359.4 |  |
| **Age at treatment** (mean(SD)) | 48.12 (15.39) | 48.12 (15.55) | <0.001 |
| **Number of infused CD34+ cells** (mean (SD)) | 3.11 (2.31) | 7.03 (3.10) | 1.435 |
| **Number of infused CD3+ cells** (mean (SD)) | 39.68 (61.48) | 297.00 (151.00) | 2.232 |
| **Sexe** = Male (%) | 114.7 (60.7) | 215.3 (59.9) | 0.016 |
| **Hematological disease group** (%) |  |  | 0.063 |
| AL | 138.2 (73.1) | 272.4 (75.8) |  |
| Lymphoid | 0 ( 0.0) | 0 ( 0.0) |  |
| Myeloid | 50.8 (26.9) | 87 (24.2) |  |
| **Disease Risk Index** (%) |  |  | <0.001 |
| low | 43.3 (22.9) | 82.3 (22.9) |  |
| int | 103 (54.5) | 195.9 (54.5) |  |
| high | 39.5 (20.9) | 75.1 (20.9) |  |
| very high | 3.2 ( 1.7) | 6.1 ( 1.7) |  |
| **CMV status** **recipient** = Positive (%) |  |  | 0.367 |
| **N** (ESS) | 117.4 (62.1) | 158.5 (44.1) |  |
| **Age at treatment** (mean(SD)) | 71.6 (37.9) | 200.9 (55.9) |  |
| **Number of infused CD34+ cells** (mean (SD)) | 111.5 (59.0) | 212 (59.0) | <0.001 |

***Abbreviations:*** *SMD: Standardized Mean Difference;* *BM: bone marrow; PB: peripheral blood stem cells; ATG: anti-thymoglobulin; AL: acute leukemia; MDS: myelodysplastic syndrome; MPS: myeloproliferative syndrome;RIC: reduced intensity conditioning; MAC: myeloablative conditioning; NMA: non myeloablative conditioning; CMV: cytomegalovirus.*

******* *”AL” include acute lymphoblastic leukemia, acute myeloblastic leukemia, biphenotypic acute leukemia, undifferentiated acute leukemia, “myeloid diseases” include myelodysplastic syndrome and myeloproliferative syndrome and "lymphoid diseases" include Hodgkin's lymphoma, non-Hodgkin's lymphoma, chronic lymphocytic leukemia and multiple myeloma.*
