# Supplementary material for: Bone marrow graft versus peripheral blood graft in haploidentical hematopoietic stem cells transplantation: a retrospective analysis in1344 patients of SFGM-TC registry
Source: J Hematol Oncol. 2024 Jan 7;17:2. doi: 10.1186/s13045-023-01515-4 (PMC10773006; doi:10.1186/s13045-023-01515-4)
Supplement: Supplementary file 4 — Additional file 4. Supplementary figures. [file 13045_2023_1515_MOESM4_ESM.docx]

**Additional file 4 : Supplementary figures**

**Cf figures file**

- Figure S1: Standardized mean differences for unweighted and weighted populations (A: BM vs. PB, B: BM vs. PB+ATG, C: PB vs. PB + ATG
- Figure S2: A: BM vs. PB in the subgroup of patients with AL and MDS/MPS with NMA conditioning, B: BM vs. PB in the subgroup of patients with AL and MDS/MPS with intensive conditioning)
